# Leucokeratosis Buccalis

**Published:** 1900-09-15

**Authors:** J. W. Leahy


### LEUCOKERATOSIS BUCCALIS.

BY J. W. LEAHY, D.D.S., M.D.

Read before the Cincinnati Odontological Society, May 25, 1900.

*Synonyms.*—Leucoplakia Buccalis, Leucoma, Psoriasis, Ichthyosis, Tylosis, Keratosis, Plaques Opalines, Smokers' Patches, etc.

*Definition and Symptoms.*—Under these various terms have been described, localized and diffused changes in which the mucous membrane of the tongue, of the cheek and exceptionally of the gums. They are unsymmetrical patches of various shapes, white or bluish white, sometimes a pearly white in color, chronic in course, and without tendency to ulcerate. The intensity of the opaque white color depends upon the thickness of the epidermis. The patches may extend and become slightly papillomatous. There are instances in which genuine epithelioma has developed from them.

*History and Symptoms.*—Mr. Hutchinson was the first to suggest the term Leucoma for the white, smooth patches while Bazin, in 1868, is credited with a description of this affection which he regarded as a psoriasis of the mucous membranes. Some years before, however, Sigmund had referred to certain syphilitic changes in the mucous membranes under the name of "Lingual Psoriasis." Schwimer used the term Leucoplakia as early as 1877. An excellent clinical description of the chronic changes in the mucous membrane of the mouth will be found in Butlin's "Diseases of the Tongue."

Schwimer described dark red spots or patches as the first manifestation of the disease. This stage is also mentioned by Barker, which Butlin, Fordyce and others who have seen many cases failed to observe. The first departure from the normal to which the patient's attention is directed consists of a bluish white or pearly colored patch or band of mucous membrane over the dorsum of the tongue. The affected patch is usually dry, rough and sharply defined from the surrounding mucous membrane. The thickened epithelium may be removed or peel off, leaving a red, smooth surface which shows some tendency to bleed. The tongue at this stage is pliable and not at all painful unless from the presence of superficial fissures. The changes in the mucous membrane may be general from the beginning, or limited to a small patch which slowly spreads in a peripheral manner, while the altered epithelium becomes thicker and more opaque.

The mucous membrane of the cheeks, corresponding to the line where teeth meet when closed, is frequently involved at the same time the disease makes its first appearance on the tongue. In the later stages of the affection fissures and furrows sometimes form, and the patient complains of stiffness and drynesss of the organ. Hypertrophy of the papillæ sometimes takes place, giving the tongue a warty appearance. If the causes which brought about the condition persist, the affected patch may be made raw and tender; otherwise it may escape the attention of the patient altogether. Marked soreness is seldom met with, unless the fissures be irritated by highly-seasoned food or alcoholic drinks. In exceptional instances certain of the patches may spontaneously disappear. A single plaque may form and remain stationery for several months or years, when rapid extension takes place, either by direct enlargement of the primary lesion or by the confluence of a number of secondary ones. The affection is interesting in this connection because tongues so affected show a great tendency to develop epithelioma. Butlin believes that the association of epithelioma has been underrated rather than exaggerated.

Epitheleomata may originate from such plaques as a warty outgrowth from their surfaces, as a hard nodule beneath the mucous membrane or an ulcer with indurated edges may be the first manifestation of the malignant process.

A difference of opinion exists as to whether the first change is in the epithelium or an inflammatory process in the papillary region. Fordyce thinks the latter view is probably correct, the hyperkeratosis being the result of impaired nutrition of the epidermis rather than the cause of the underlying cell infiltration.

The microscope reveals a marked thickening of the horny layer, the cells of which still preserve their nuclei. In places the papillæ are almost obliterated, exceptionally however, the interpapillary processes show evidences of proliferation. There is always an inflammatory infiltration in the derma. Leloir's investigations showed that epitheliomata did not develop directly from the surface affected with hyperkeratosis, but from the ulceration resulting from the desquamation of the diseased epithelium from secondary fissures and other lesions.

In a few cases of epithelioma in this connection, examined by Fordyce, the new growth belonged to tubular epitheliomata, with little or no tendency to the formation of horny tissue.

This affection is almost exclusively confined to the male, and generally occurs after middle life. It may exceptionally be met with in women, and occur as early as the twenty-first year. The combined influence of tobacco smoke and syphilis are the most potent agents in its etiology; either may produce it, or it may exceptionally occur in individuals who are not users of tobacco and who have never had syphilis. Undiluted alcoholic drinks, highly seasoned foods and irritation from rough teeth or plates have some influence in causing its development in predisposed persons.

The irritation of early syphilitic lesions of the mouth by smoking and drinking is undoubtedly one of the most important etiological factors in its production, the resulting leucokeratosis being rather the result of the disease than syphilitic, *per se*.

Prognosis is somewhat doubtful unless the case is seen early in its development and the patient can be persuaded to abstain from the use of tobacco and irritating food and drink. There are abortive forms which spontaneously disappear, and, in a certain percentage of cases, no tendency to the warty outgrowth or to the development of epithelioma takes place. The possibility of the occurrence of malignant disease should always be borne in mind.

*Treatment.*—The use of tobacco in every form is to be prohibited absolutely; alcohol should be given up. Acids, sweets, highly seasoned foods, as well as all articles which are too hot or too cold, should be interdicted. Every source of local irritation should be removed and strict attention paid to the hygiene of the mouth. Very little can be done in the majority of instances by constitutional treatment; much may be done by local means, if not to cure the disease, yet certainly to relieve it. In the lighter cases the patient will probably do nothing more than wash the mouth with solutions or gargles. He may use a solution of twenty grains of bicarbonate of potash to an ounce of water. If the patient has had syphilis, a weak solution of chromic acid, one or two grains to an ounce of water, if used as a wash, or five to ten grains if used as an application, in more severe cases. The tongue should be painted or washed frequently with an alkaline wash, such as a solution of bicarbonate of soda, or a solution of borax, or a weak solution of alum, two grains to the ounce of water. One general rule holds good in all cases, viz., not to use caustics, as they may do harm, and whatever danger there may be of the development of carcinoma is certainly increased by the employment of nitrate of silver or other caustics. A small and sharply defined patch can be removed by the knife, and success may result from the galvano cautery when the disease is more extensive.

*Report of Case.*—Mr. J. C., age thirty-five; occupation, coffee-roaster; sent to me January 15, 1900, by his physician. He complained that for some time he could not take any food very hot or cold, sweet or sour. No history of syphilis, was not a very heavy smoker, but held a pipe in his mouth most of the day, also was in the habit of eating the hot roasted coffee when finished browning it; at times it hurt his tongue very much. The left side of the tongue appeared more sensitive than the right—more patches, and it seemed to be more inflamed. He held the pipe on the left side of the mouth. When the tongue was protruded I noticed the entire surface was deprived of its natural color; instead of red it was a bluish white, quite smooth except where it was furrowed here and there. I requested him to give up smoking, to be very careful not to take any food very hot or cold, and prescribed a bicarbonate of soda wash; painted the tongue every day with a 2 percent solution of chromic acid. There is a marked improvement in the tongue now, and it is coming back to its normal color, not as fast as I would like, still it is doing very well. I shall report later on how I succeed with it.

### DISCUSSION.

Dr. Stern: I read recently in a German journal a statement by Dr. Schweimer that this disease is most readily contracted by persons in whose mouths there is an unusual thinness of the epithelium. He cites syphilis as the necessary cause. Alkaline mouth washes are recommended in these cases. Chromic acid has been used to effect a cure. Paint the affected membrane with a 2 percent to 4 percent solution. Smoking is not especially detrimental. High seasoning of food is supposed to aggravate the condition.

Dr. Hunter: Have you seen many cases of this disease? It is something entirely new to me. I have seen many diseases of the tongue, but nothing answering the description of this disease.

Dr. H. A. Smith: Dr. Hunter's question suggests that dentists should know more than they do about the tongue and its diseases. This is not an uncommon disease; it occurs frequently; not being close observers, or having no knowledge of it, we have not identified it. I would not like to affirm that it properly comes within the province of the dentist to treat it; as stomatologists we might. The authorities quoted to-night are the best I have any knowledge of on tongue diseases. A singular appearance of the tongue in this disease has attracted my notice; it looks like white paint become discolored and dirty. It is a matter of importance when the true nature of this affection is recognized not to alarm the patient by declaring its serious nature.

Dr. Hunter: That sounds like the doctrine of the faith curists.

Dr. Smith: I am not one of that class, but I maintain that a state of mental tranquility is most favorable to the cure of disease, especially malignant disease. When Gen. Grant died a great many persons came to us to have their teeth examined, being alarmed by the report that his cancer was due to the irritation of a broken tooth.

I do not understand why this particular disease is not oftener induced by the condition of artificial teeth, as we so often find them. Loose teeth moving about and impinging on the soft tissues as they so frequently do, might be expected to provoke this trouble.

My own belief as to the treatment of this disease is that it should be alkaline. I should hesitate to use chromic acid. McKesson & Robbins preparation of glyco-thymoline is an excellent preparation for treating this condition.

Dr. Cassidy: I can recall only two cases of leucoma. One said to be due to smoking, the other to a loose plate. In my opinion, this is not so much a disease as a symptom of a disease. The local manifestation is due to some irritation in the mouth. The disease itself, I think, is hardly due to smoking. Women have it as well as men. The systemic condition, according to my reading on the subject, is said to exhibit an alteration of the condition of the red blood corpuscles. I can not agree with the opinion of the writer of this paper that systemic treatment is not indicated. Iron and quinine, it seems to me, are indicated; quinine to prevent the migration of the white corpuscles.

Dr. Heise: Dr. Hunter says he has never seen a case of this disease. He may have overlooked the disease, as it is not always troublesome; dentists do not observe all the buccal tissues closely enough. The college clinics are good places to study these unusual diseases If the dental colleges do not attempt to treat these diseases they would confer a great benefit on the profession as well as upon individual dentists, by following them up and reporting the outcome of any treatment made. The German dental colleges, being government institutions, are required to do this. In regard to the frequency of this disease, Schweimer states that in one thousand cases of syphilis he meets three or four cases of leucoma, and that in five thousand cases he examined for leucoma, and found it twenty times. From 1878 to 1890 he noted 220 cases of leucoma. He claims that these cases, if taken in time and properly managed, are as amenable to treatment as many other diseases. He does not inhibit smoking in moderation; he has great confidence in the application of papayotine.

I do not approve of alkaline washes for this disease. On general principles I think alkaline preparations are contra-indicated in all mouth treatment. I certainly will not use free alkaline washes in my practice—such, I mean, as bicarbonate of soda. Caustic alkali is liable to result, especially if hard water be used.

Dr. Leahy: This, like many other diseases of the mouth, is more common than some of us think. As has been said, we are not always as observant as we need to be. We should know enough to at least recognize these various diseases as they are presented to our notice, since the patient often is wholly unconscious of any real danger. We may save him serious trouble if we are able at least to refer him to the surgeon for treatment when we find such treatment out of our province.

